# Adoption of a dedicated multidisciplinary team is associated with improved survival in acute pulmonary embolism

**DOI:** 10.1186/s12931-020-01422-z

**Published:** 2020-06-22

**Authors:** Lukasz A. Myc, Jigna N. Solanki, Andrew J. Barros, Nebil Nuradin, Matthew G. Nevulis, Kranthikiran Earasi, Emily D. Richardson, Shawn C. Tsutsui, Kyle B. Enfield, Nicholas R. Teman, Ziv J. Haskal, Sula Mazimba, Jamie L. W. Kennedy, Andrew D. Mihalek, Aditya M. Sharma, Alexandra Kadl

**Affiliations:** 1 Department of Medicine, Division of Pulmonary and Critical Care, University of Virginia, Charlottesville, USA; 2 Department of Medicine, University of Virginia, Charlottesville, USA; 3 Department of Surgery, Division of Thoracic and Cardiovascular Surgery, University of Virginia, Charlottesville, USA; 4 Department of Radiology and Medical Imaging, Division of Vascular and Interventional Radiology, University of Virginia, Charlottesville, USA; 5 Department of Medicine, Division of Cardiovascular Medicine, University of Virginia, Charlottesville, USA; 6 Department of Pharmacology, University of Virginia, Charlottesville, VA USA

**Keywords:** Acute pulmonary embolism, Pulmonary embolism response team, PERT, Acute pulmonary embolism interventions

## Abstract

**Background:**

Acute pulmonary embolism remains a significant cause of mortality and morbidity worldwide. Benefit of recently developed multidisciplinary PE response teams (PERT) with higher utilization of advanced therapies has not been established.

**Methods:**

To evaluate patient-centered outcomes and cost-effectiveness of a multidisciplinary PERT we performed a retrospective analysis of 554 patients with acute PE at the university of Virginia between July 2014 and June 2015 (pre-PERT era) and between April 2017 through October 2018 (PERT era). Six-month survival, hospital length-of-stay (LOS), type of PE therapy, and in-hospital bleeding were assessed upon collected data.

**Results:**

317 consecutive patients were treated for acute PE during an 18-month period following institution of a multidisciplinary PE program; for 120 patients PERT was activated (PA), the remaining 197 patients with acute PE were considered as a separate, contemporary group (NPA). The historical, comparator cohort (PP) was composed of 237 patients. These 3 groups were similar in terms of baseline demographics, comorbidities and risk, as assessed by the Pulmonary Embolism Severity Index (PESI). Patients in the historical cohort demonstrated worsened survival when compared with patients treated during the PERT era. During the PERT era no statistically significant difference in survival was observed in the PA group when compared to the NPA group despite significantly higher severity of illness among PA patients. Hospital LOS was not different in the PA group when compared to either the NPA or PP group. Hospital costs did not differ among the 3 cohorts. 30-day re-admission rates were significantly lower during the PERT era. Rates of advanced therapies were significantly higher during the PERT era (9.1% vs. 2%) and were concentrated in the PA group (21.7% vs. 1.5%) without any significant rise in in-hospital bleeding complications.

**Conclusions:**

At our institution, all-cause mortality in patients with acute PE has significantly and durably decreased with the adoption of a PERT program without incurring additional hospital costs or protracting hospital LOS. Our data suggest that the adoption of a multidisciplinary approach at some institutions may provide benefit to select patients with acute PE.

## Background

Acute pulmonary embolism (PE) remains a significant cause of mortality and morbidity worldwide, and is the third most common cause of death among hospitalized patients [1]. Still, improving trends in mortality and other patient-centered outcomes have been reported and seem to track with shorter time-to-diagnosis as well as increasing use of low-molecular-weight-heparin, direct oral anticoagulants and more advanced therapies such as thrombolysis, surgical embolectomy and catheter-based interventions [2]. Prompted in part by the lack of clear guidelines surrounding the use of these emerging, advanced treatment modalities, the adoption of a multidisciplinary approach to management of acute PE has increased in popularity across academic centers. Such teams have been assembled as a reasonable response to address often complex clinical situations and varying institutional experiences with these therapies. The rapid nationwide growth of these teams has been catalyzed by the recent formation of the National PERT consortium. This trend was most recently acknowledged by the 2019 European Society of Cardiology (ESC) guidelines, which published a class IIA recommendation promoting institutional establishment of PERTs for the purpose of managing high-risk and selected cases of intermediate-risk PE, a recommendation based on the lowest level of evidence (C) [3].

Early published reports of single-center experiences with PERT programs have been useful in describing trends in management but did not specifically evaluate the effect of these teams on patient-centered outcomes [4–6]. Evaluating the efficacy of multidisciplinary PE teams in improving patient-centered outcomes is complex as contemporaneous intra-institutional outcomes analyses have been seen as not feasible. Randomized, controlled trials have also been precluded given concerns over clinical equipoise and perceived consent requirements which could hamper adequate enrollment. Although comparisons to historical outcomes have been published, only one recent single center study reported a significant reduction in mortality [7–9]. The absence of clear, durable benefits that can be attributed to PERTs is striking as long-term trends alone during this time period have indicated improving outcomes. In the absence of supportive outcome analyses of this intervention, it is difficult to ascertain whether the institutional costs and educational efforts necessary to develop such a robust infrastructure, which draws heavily on institutional resources and personnel, may be more meaningfully allocated elsewhere.

To address these important questions, we undertook an outcomes analysis of an 18-month period of our operating PERT program, comparing these outcomes to both historical and contemporary cohorts.

## Methods

### Organization and structure of the PE response team

The University of Virginia Medical Center (UVAMC) Multidisciplinary PERT was formally inaugurated in March 22, 2017 and consists of specialists from cardiology, pulmonology, vascular medicine, interventional radiology, hematology, cardiac surgery and pharmacy. In 2016, Massachusetts General Hospital published initial data on a pulmonary embolism response team that rapidly engaged multiple disciplines to deliver efficient, organized care to PE patients [4]. In order to establish a multidisciplinary PE response team at UVA after the same model we 1) identified key team members, 2) provided in-house education on the reason for and role of the PE-response team, 3) developed a standard work with our call center to guide the activation of the team, 4) established uniform consult note templates to ensure uniformity and support quality control, and 5) ensured follow up after discharge for all PE alerted patients. Formal activation criteria required a patient to have CT pulmonary angiogram confirming acute pulmonary embolism with evidence of right heart strain (by imaging or elevation of cardiac biomarkers [BNP and/or troponin]) and/or hypotension.

### Data collection

We conducted a single-center retrospective cohort study at the University of Virginia Medical Center to evaluate outcomes and treatment trends before and after implementation of a multidisciplinary PE response team at our institution. All PERT-activations starting March 22, 2017 through October 21, 2018 were collected in a prospective manner as part of a quality assurance initiative. Subsequently, 18-month data starting April 22, 2017 were analyzed, which allowed for a 1-month acclimation of our institutional practice. We queried our clinical database warehouse for all encounters during this same 18-month period meeting search criteria of a billing diagnosis of PE and available computed tomography pulmonary angiogram and new inpatient or discharge prescription for an anticoagulant during the same period, excluded encounters during which PERT was activated, and designated this group as the contemporary, non-PERT-activated (NPA) group. Our historical, pre-PERT (PP) cohort was similarly composed of consecutive patients treated for acute PE at our institution from July 2014 through June 2015. Across these 3 mutually exclusive groups, data on basic demographics, comorbidities, metrics informing PE risk categories (PE severity index [PESI] and European Society of Cardiology [ESC]) and severity, PE risk factors, treatment, mortality and post-hospital follow-up were extracted from the electronic medical record. Institutional Review Board approval for publication of the analyses of these data was obtained.

### Outcomes

Our primary outcome was all-cause mortality among patients with confirmed acute PE, which was assessed using the Kaplan-Meier method to estimate the survival function of the cohorts. Additional outcomes analyzed included total hospital charges, hospital length-of-stay (LOS), 30-day readmissions and in-hospital bleeding rates. The Vizient database was used to retrieve data on expected length-of-stay based and reported total hospital charges.

### Statistics

Kaplan-Meier curves were constructed for analysis of survival between cohorts in aggregate and in an intervention-stratified manner. Pearson’s chi-squared tests and Fisher’s exact tests were used to analyze prevalence of risk markers between groups. Ordinary one-way ANOVA was used to compare LOS data and the Kruskal-Wallis test was employed to compare hospital charge data. *P* < 0.05 was considered statistically significant.

## Results

**Fig. 1:**
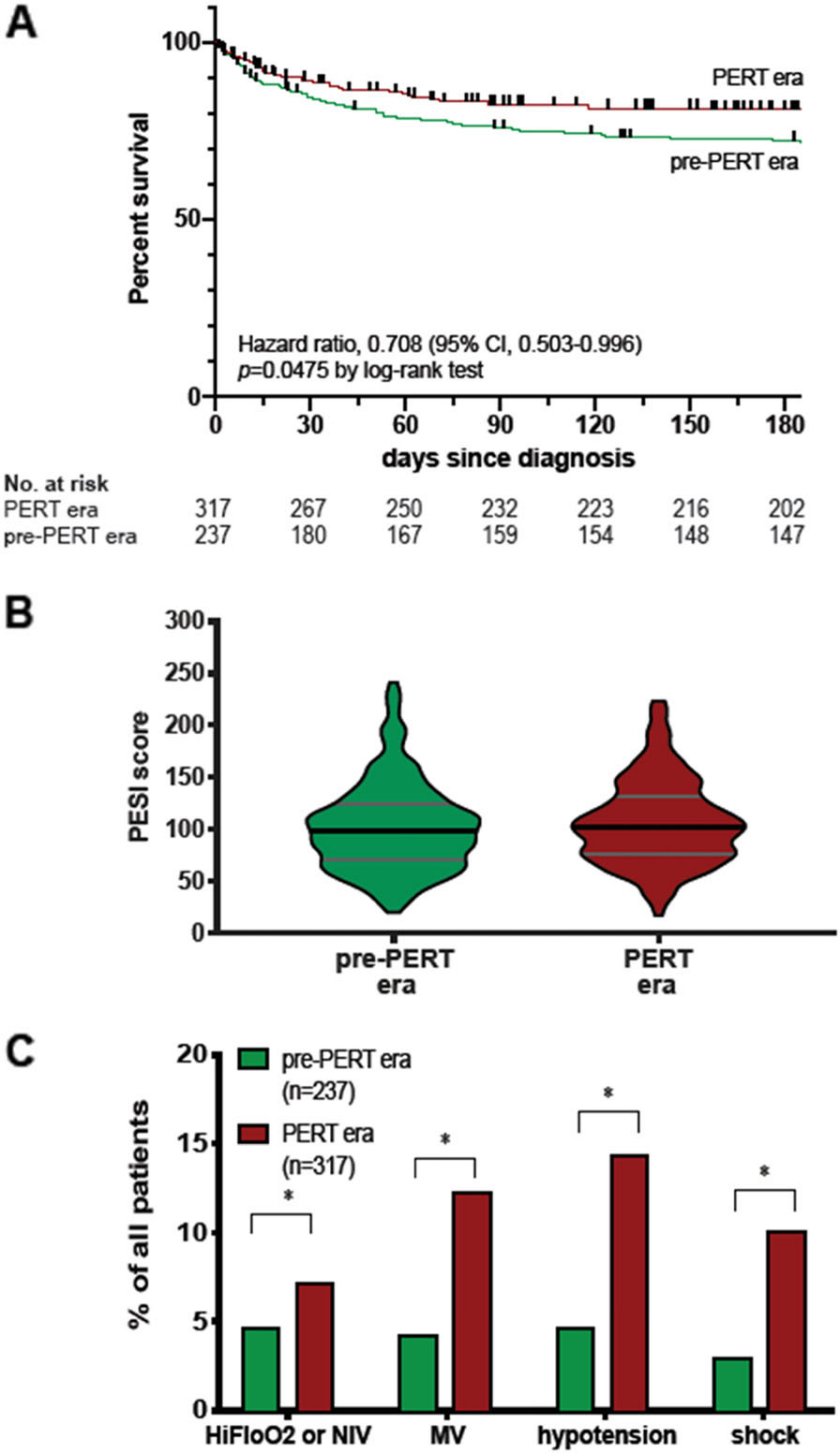
Kaplan-Meier survival curve of pre-PERT-era and PERT-era cohorts (**a**) and prevalence of severity of markers among the 2 cohorts (**b**). Hi-Flo, high-flow oxygen nasal cannula (> 15 L per minute); MV, mechanical ventilation, hypotension is defined as systolic blood pressure between 100-110mmHG and 40 mmHg lower than at baseline; shock is defined as hypotension requiring vasopressor medication. * *p* = < 0.05 by Chi square test

The UVAMC PERT-era cohort included 317 consecutive patients with confirmed acute PE starting April 22, 2017 through October 21, 2018. During this period there were 124 PERT activations, of which 120 had a confirmed PE. The remaining non-PERT activated (NPA) group consisted of 197 patients. The PP historical cohort was composed of 237 consecutive patients with diagnosis of acute PE from July 2014 through June 2015. These 2 cohorts were statistically similar in terms of baseline demographics, comorbidities and PESI scores (Table 1). Kaplan-Meier survival analysis of patients stratified by cohort demonstrated significantly improved survival time-from-PE diagnosis in the PERT-era cohort when compared with patients treated during the pre-PERT era (Fig. 1a; *p* = 0.0475; survival 89.6% vs. 84.8% at 30 days and 81.4% vs 75.9% at 180 days). The prevalence of PE severity markers not captured by PESI, including requirement for high-flow nasal cannula oxygen, mechanical ventilation and shock requiring vasopressor support, were all more prevalent during the PERT-era (Fig. 1b; *p* < 0.05 for all comparisons).

**Table 1:** Baseline Characteristics

	pre-PERT (PP) (***n*** = 237)	PERT-alerted (PA) (***n*** = 120)	Non-PERT alerted (NPA) (***n*** = 197)	***P*** value
Age, median (95%CI)	62 (59–66)	63.5 (60.0–67)	62 (58–63)	n.s.
Sex, No. (%)				
Men	124 (52)	60 (48)	104 (53)	n.s.
Women	113 (48)	60 (50)	93 (47)	n.s.
BMI, mean (SD)	30.5 (9.17)	32.35 (9.08)	31.4 (9.00)	n.s.
PESI score, median (95%CI)	108 (102–114)	102 (95–109)	103 (94–107)	n.s.
PESI score, mean (SD)	114 (48.63)	109 (38.79)	105 (41.36)	n.s.
PESI classes, No. (%)				
PESI class I	37 (16)	10 (8)	36 (18)	n.s.
PESI class II	34 (14)	27 (23)	38 (19)	n.s.
PESI class III	39 (16)	27 (23)	32 (16)	n.s.
PESI class IV	48 (20)	22 (18)	35 (18)	n.s.
PESI class V	79 (33)	34 (28)	55 (28)	n.s.
Coexisting conditions, No. (%)				
Heart failure	92 (39)	6 (5.0)	20 (10)	< 0.001
Chronic lung	59 (25)	11 (9)	34 (17)	0.001
Malignancy	79 (33)	43 (36)	72 (37)	0.747
Previous VTE	44 (19)	31 (26)	44 (22)	0.266

*Abbreviations*: *PP* pre-PERT, *PA* PERT alerted, *NPA* Non-PERT alerted, *BMI* Body Mass Index, *PESI* Pulmonary Embolism Severity Index, *VTE* Venous Thromboembolism. *P* value calculated by Chi Square test

**Fig. 2:**
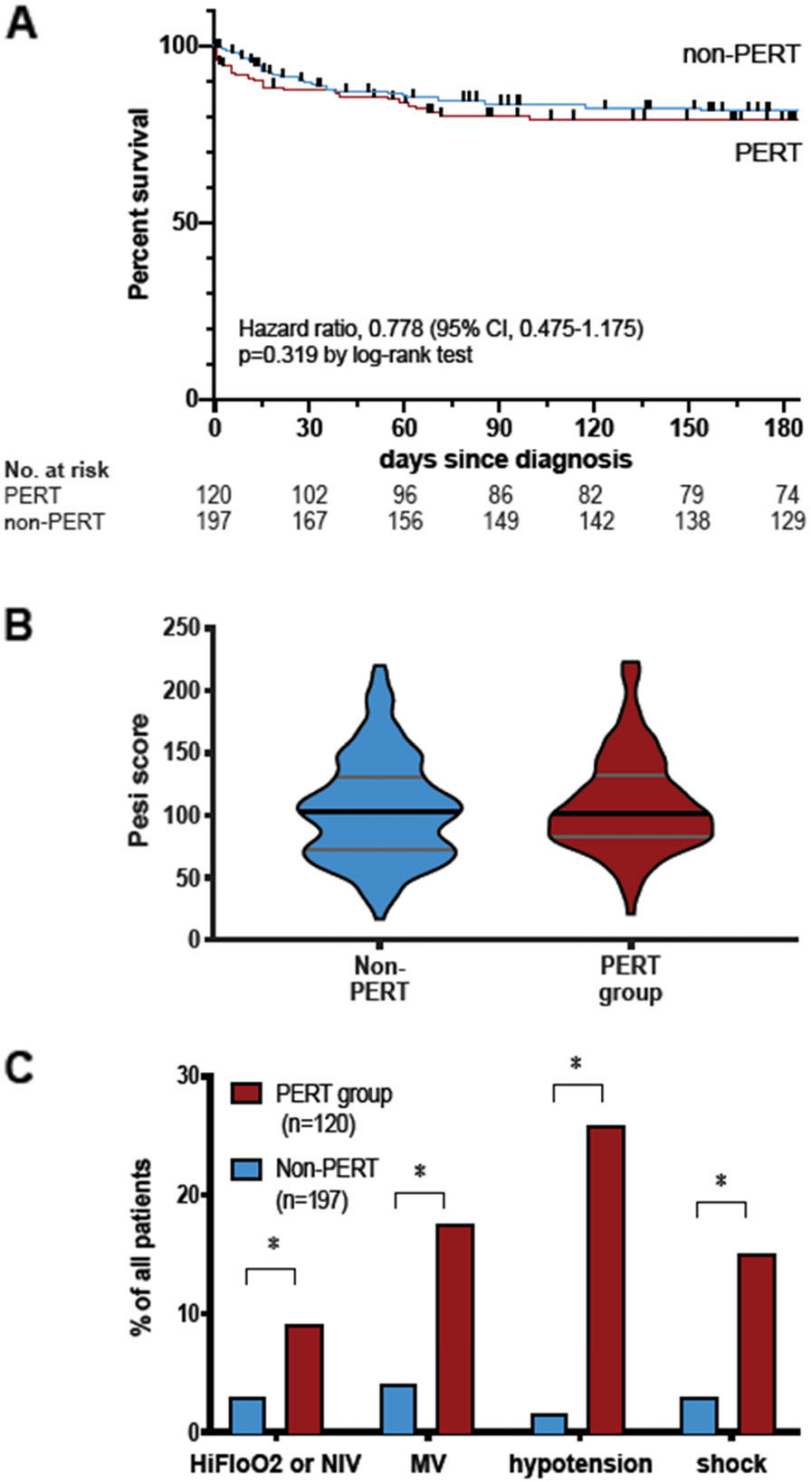
Kaplan-Meier survival curve of PERT-alerted and non-PERT-alerted patients during the PERT-era (**a**) distribution of PESI scores in the 2 groups (**b**) and prevalence of severity of markers among the 2 groups (**c**). Hi-Flo, high-flow oxygen nasal cannula, high flow oxygen (> 15 L per minute); MV, mechanical ventilation, hypotension is defined as systolic blood pressure between 100-110mmHG and 40 mmHg lower than at baseline; shock is defined as hypotension requiring vasopressor medication. * *p* < 0.001 by Chi square test

During the PERT era, Kaplan-Meier estimates did not demonstrate any statistically significant difference in survival time in PA patients when compared to NPA patients (Fig. 2a, survival 87.5% vs 90.9% at 30 days and 80% vs 82.2% at 180 days). Although patients in these 2 contemporary groups had similar PESI scores, the prevalence of severe hypoxia requiring either oxygen via high-flow nasal cannula or mechanical ventilation and relative hypotension or shock requiring vasopressor support were all significantly and markedly more prevalent in the PA group (Fig. 2b and c; *p* < 0.05 for all comparisons in 2c). ESC risk categories in the PA group are provided in the supplement. Comparative, ESC category-stratified analyses could not be performed due to missing data in the both PP and NPA patients. We observed that transthoracic echocardiography, troponin and BNP were available in 44, 48 and 31% of PP patients, respectively. We observed that these stratification markers were obtained at significantly higher rates during the PERT-era, even in NPA patients (Table online).

**Fig. 3:**
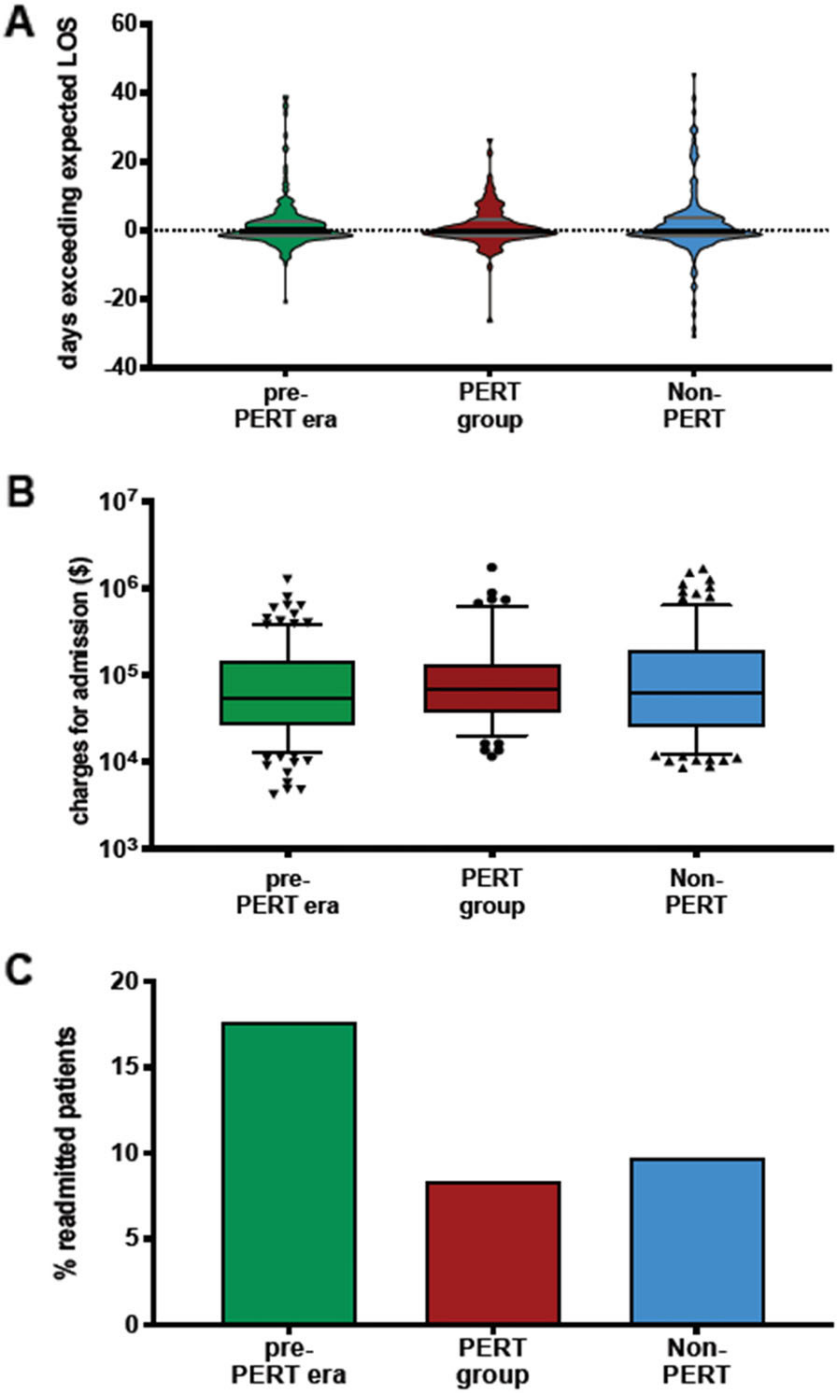
Distribution of days exceeding expected hospital LOS, Length of stay (**a**), total costs for the admission charged, and rate of 30-day readmission (**c**). (A, *p* = 0.8475; B, *p* = 0.185; C, *p* = 0.047 by Fisher’s exact test). PP; pre-PERT; PA, PERT alerted; N-PA, Non-PERT alerted

Normalized hospital length-of-stay (LOS) did not significantly differ in the PA group when compared to either the NPA or PP groups (Fig. 3a, *p* = 0.8475) and no difference in hospital costs was observed among the 3 groups (Fig. 3b, *p* = 0.185). However, 30-day hospital readmission rates were significantly lower in the PERT era (Fig. 3c, *p* = 0.047).

Rates of advanced therapies were significantly higher in the PERT era (9.1% vs. 2%) and were concentrated in the PA group (21.7% vs. 1.5%, Table 2). Specifically, 17 patients in the PA group underwent catheter-directed thrombolysis with another 5 patients receiving CD-thrombectomy. An additional 5 and 2 patients received systemic thrombolysis and surgical thrombectomy, respectively. This increased utilization of advanced therapies was not attended by any significant rise in in-hospital bleeding complications (Table 2). The use of IVC filters was significantly lower in the PERT era with 22% of patients receiving filters in the pre-PERT era, 16% in the PA group and 11% in the NPA group (*p =* 0.008). Compared to the PP group, contemporary rates of warfarin use declined with increasing use of novel oral anticoagulant use (Table 3). Finally, dedicated outpatient follow-up of acute PE was achieved in 58% of PA patients alive at hospital discharge (data not shown).

**Table 2:** PE management and bleeding complication

	pre-PERT (PP) (n = 237)	PERT-alerted (PA) (n = 120)	Non-PERT alerted (NPA) (n = 197)	***P*** value
AC only, No. (%)	224 (95)	81 (68)	174 (88)	< 0.001
IVC filter, No. (%)	53 (22)	19 (16)	22 (11)	0.008
Any advanced, No. (%)	5 (2)	26 (22)	3 (2)	< 0.001
Cath directed thrombolysis	3 (1)	17 (14)	1 (1)	< 0.001
Cath directed thrombectomy	4 (2)	5 (4)	3 (2)	0.245
Systemic lysis	0	5 (4)	0	< 0.001
Surgical thrombectomy	0	2 (2)	0	0.047
ECMO	0	3 (3)	1 (1)	0.016
Bleeding complication, No. (%) ^a)^				
Minor	15 (6)	9 (8)	20 (10)	0.340
Major	9 (4)	1 (1)	4 (2)	0.260

*Abbreviations*: *PP* pre-PERT, *PA* PERT alerted, *NPA* Non-PERT alerted, *AC* anticoagulation, *IVC* Inferior Vena cava, *ECMO* Extracorporeal membrane oxygenation. ^a)^ Bleeding complication severity assessed by the International Society on Thrombosis and Haemostasis. *P* value calculated by Fisher’s Exact test

**Table 3:** Anticoagulation at discharge

	pre-PERT (PP) (***n*** = 215)	PERT-alerted (PA) (***n*** = 107)	Non-PERT alerted (NPA) (***n*** = 185)	***P*** value
none, No. (%)	21 (10)	7 (7)	14 (8)	0.581
Apixaban, No. (%)	76 (35)	71 (66)	105 (57)	< 0.001
Dabigatran, No. (%)	0	2 (2)	5 (3)	0.032
Edoxaban, No. (%)	0	1 (1)	2 (1)	0.322
Enoxaparin, No. (%)	34 (16)	16 (16)	28 (15)	0.987
Enoxaparin+Warfarin, No. (%)	0	6 (6)	0	< 0.001
Fonadaparinux	1 (1)	0	1 (1)	1.000
Rivaroxaban, No. (%)	37 (17)	2 (2)	12 (7)	< 0.001
Warfarin, No. (%)	48 (22)	2 (2)	18 (10)	< 0.001

*Abbreviations*: *PP* pre-PERT, *PA* PERT alerted, *NPA* Non-PERT alerted. Only patients discharged alive from the hospital were included. *P* value calculated by Fisher’s Exact test

## Discussion

In our single-center retrospective cohort analysis of 554 admitted patients treated for acute PE we report that PERT era survival of patients was significantly improved compared to a recent historical cohort with similar disease severity. While the improved outcomes may be attributable to a secular trend characterized by a growing reliance on novel and advanced therapies, the recommendation and coordination of such therapies by a dedicated PERT may have mediated this effect.

While multiple studies have detailed single-center experiences of PERT institution, only four studies have attempted to analyze patient-centered outcomes attending implementation of a PERT program. We acknowledge that all but one of these previous publications reporting on mortality in the PERT era did not demonstrate significant improvement in survival following implementation of a PERT. One possible explanation for these discordant findings is that two of the prior studies compared only PERT-activated patients to artificially constructed comparator groups defined as either historical patients that would have met criteria for PERT activation or patients with matched diagnosis codes. Specifically, Rosovsky and colleagues reported no improvement in 30-day mortality on the outcomes of PERT-activated patients compared to a historical cohort of patients that met PERT activation criteria. However, these authors acknowledge missing 30-day data for nearly half of their historical group [7]. In a more recent paper, Xenos et al. likewise reported no mortality benefit with PERT activation. In that study, the comparator group was derived from billing and diagnostic codes rather than clinical data which can furnish higher resolution disease severity analysis [8]. Such comparator groups fail to account for patients that receive the PERT intervention for complex clinical reasons that are difficult to capture with conventional risk assessment models. This practice is reflected by the similar PESI scores of our PERT-activated and non-PERT-activated patients as well as the relatively high amount of “low-risk” PE patients that are evaluated by PERT teams and are characterized by an unexpectedly high mortality [4].

Our analysis of consecutive patients treated for acute PE in 2 distinct eras eliminates the bias introduced by construction of artificial comparator groups. In a third study reporting on survival pre- and post-PERT, Jen et al. evaluated only in-hospital mortality, which is a relatively short time scale and may not reflect the true survival benefit which appears to accrue with time as demonstrated in our analysis [10].

Most recently, Chaudhury and colleagues reported on a significant 30-day mortality improvement attending the adoption of a PERT at the Cleveland Clinic [9]. Unlike previous publications, these authors did report on consecutive patients treated for acute PE and utilized a similar unselected comparator group, thereby minimizing selection bias. Using a similar study design, our findings support improved survival in the PERT era, and suggest that this benefit is durable. Our data also extend previous findings, demonstrating that PERT activation is not attended by any significant increments in LOS or hospital charges. Uniquely, our study design allowed for parsing of mortality differences attributable to secular trends (which have been reported) and those potentially attributable to institution of a PERT program. To investigate the magnitude of the survival benefit that could be attributed to the PERT intervention, we took advantage of the naturally expected “misses” (i.e. patients that met criteria for PERT activation but were not alerted as such) during the inaugural period of our PERT program to compare outcomes in contemporary populations of PERT-activated and non-PERT-activated patients. This analysis revealed statistically similar short-term survival in PERT-activated and non-PERT-activated patients, despite increased severity of disease in the PERT-activated group as assessed by severity markers not captured by PESI risk score. Mirroring practice changes reported by others, PERT era treatment at our institution relied more heavily on advanced therapies. Of our PERT-activated patients, 68% received anticoagulation alone, while 22% received some form of advanced therapy. These numbers are comparable to those reported in a recent analysis of 8 U.S. PERTs [11]. Still, there remains a great deal of heterogeneity among institutions that have adopted the PERT model, and although, in aggregate, advanced therapies may increase with PERT, this is not a necessary outcome. As previously reported, institutional experience seems to play a role in these inter-institutional differences [12].

Although we do report markedly higher rates of advanced therapies in our PERT-activated patients, we do not reach any conclusion as to how much of the salient effect of the PERT intervention is mediated by increased use of those therapies, rather than by a more personalized treatment generally, lower IVC filter placements rates, the educational efforts that accompanied our program or decreased diagnosis to treatment times as previously reported [13]. Prior, prospective trials of catheter-directed lysis have demonstrated benefit with respect to intermediate markers of disease severity, including right ventricle to left ventricle ratios and mean pulmonary artery systolic pressure [14, 15]. However, it is not known whether these intermediate, surrogate endpoints translate into improved survival. If, in fact, improved survival attributed to PERT is partly mediated by higher utilization of advanced methods, our data seem to indicate that the PERT may be an effective way to triage patients with acute PE to advanced therapies in the absence of published guidelines on their use. Still, it is highly implausible that improved survival in the PERT era could be exclusively, or even largely attributed to an additional 24 advanced therapies in the PERT era. Rather, institution-wide educational programs, decreased time to anticoagulation and the collaborative and timely multidisciplinary conferences all of which affect a greater swath of patients with acute PE may in fact be the key salient features of such programs [9].

Perhaps most interestingly, the similar survival observed in PERT-activated and non-PERT-activated patients during the PERT era raises the interesting prospect of expanding the purview of current PERT programs to potentially include lower risk patients who may stand to benefit in the short and long term with individualized therapies. The alternative being that there exists a “ceiling” to the survival benefit that can be derived by such means. In this respect, it must be noted that the rationale for some advanced therapies is deliberate in its long-term scope, which concerns are not captured by short term mortality analyses, and cannot be analyzed at the current stage of PERT adoption. Perhaps as an indirect surrogate portending improved long term outcomes, the high rates of dedicated hospital follow-up attained with PERT may both cater to data collection programs as well as to improved long term outcomes especially as related to long term management of anticoagulation, mitigation of recurrent VTE risk and development of chronic thromboembolic pulmonary hypertension [16].

In spite of an increased reliance on advanced therapies, neither in-hospital bleeding rates, LOS, nor hospital costs were appreciably different. These findings are in accord with previously published data. Across studies, an increase in bleeding rates has not been observed despite increased utilization of therapies thought to carry a higher risk of bleeding [7, 10]. This lower bleeding rate may be attributable to better patient selection, less use of heparin infusions and warfarin. In our study we also found a significantly lower rate of 30-day hospital readmission, which certainly has potential to positively affect the economic burden of acute PE. Although hospital costs do not appear to be significantly affected by incorporation of a multidisciplinary team (and resultant implementation of its recommendations), there is little doubt as to the less quantifiable but notable costs related to implementation of such a program in the first place - financial, educational, time and otherwise.

We deliberately excluded analysis of patients in the immediate pre-PERT era (2016) as efforts to launch a PERT program were underway at that time. These early efforts resulted in numerous, albeit informal, multidisciplinary care plans that were not captured in any systematic way and may have confounded a comparison of outcomes during that time period with outcomes during our formal PERT-era. We suggest that such early efforts may also confound the analysis of others. Additionally, risk-stratified survival analysis by ESC categories was hampered due to lower rates of echocardiography and cardiac biomarker acquisition, precluding accurate categorization of non-high-risk patients in our historical and non-PERT-activated groups. Transthoracic echocardiography, troponin and BNP were available in 44, 48 and 31% of historical patients, respectively. While these rates compare with those reported in the EMPEROR registry data [17], these stratification markers were obtained at significantly higher rates during the PERT-era, even in non-PERT-activated patients.

Finally, it is difficult to capture the effect that such an intervention may have on family and patient care partners even if the eventual outcome is unavoidably poor. As has been observed with rapid response teams generally, team members are often uniquely involved in end-of-life care. While engaging end-of-life issues may not be the stated purpose of such teams, these issues should be approached with some intentionality, especially in the acute PE population characterized by a high prevalence of terminal cancer diagnoses [18]. The comfort of knowing that all institutional resources and expertise have been brought to bear on the care of an individual patient may relieve lingering regret experienced by family members of deceased loved ones and significantly improve patient and family satisfaction.

Our study has several limitations. In the first place, our study was not a prospective, randomized trial, therefore we cannot attribute improved outcomes to the PERT intervention explicitly. As previously discussed, such trials are unlikely given concerns over clinical equipoise and adequate enrollment. Secondly, as a single-center retrospective analysis, PERT-activated patients clearly differ from non-alerted patients in both PE severity markers and, presumably, ways that cannot be reduced to a simple risk score or index and many PERT activations for intermediate risk PEs may be driven by intangible appreciation of particular clinical scenarios. Finally, our extensive institutional experience with minimally-invasive interventional methods, such as catheter-based therapies, may limit the generalizability of our conclusions to other centers with similar experience and infrastructure.

## Conclusions

At this time, current VTE management guidelines do not provide clear guidance on the appropriateness of many advanced therapies [3, 19, 20]. Additionally, only a weak recommendation has been made regarding the development or adoption of multidisciplinary rapid response teams for acute PE. Our data suggest that the adoption of a multidisciplinary approach at some institutions may provide benefit to select patients with acute PE.

## Supplementary information

**Additional file 1: Supplemental Figure 1.** Distribution of 2019 ESC risk categories among the 3 groups. ESC, European Society of Cardiology.

**Additional file 2: Supplemental Figure 2.** Prevalence of non-PE related mechanical ventilation requirement and shock among patients among (A) pre-PERT and PERT-era patients and (B) Pert activated and non-PERT activated subjects in the PERT era; MV, mechanical ventilation, shock is defined as hypotension requiring vasopressor medication. * *p* < 0.001 by Chi square test.

**Additional file 3: Supplementary Table 1** Performed risk assessment and severity of PE. Abbreviations: PP; pre-PERT; PA, PERT alerted; NPA, Non-PERT alerted; BNP, Brain natriuretic peptide; TTE, transthoracic echocardiogram; RV, right ventricle; CT, computer tomography. a) assessment within 12 h of pulmonary embolism diagnosis, b) positive RV strain by biomarkers was determined as troponin> 0.02 ng/mL, and/or BNP > 155 pg/mL; c) RV strain on CT imaging was determined as RV/LV ration (ratio of right ventricular to left ventricular diameter ration of > 0.9). *P* value calculated by Chi Square test.

## Data Availability

Vizient database was employed in some analyses, this database is publicly available. Additional supplemental data as referenced in the manuscript will be made publicly available online. The dataset analyzed in the current study is available from the corresponding author on reasonable request.

## Abbreviations

AC: Anti-coagulation
ECMO: Extra-corporeal membrane oxygenation
IVC: Inferior vena cava
MV: Mechanical ventilation
NPA: non-PERT alerted
PA: PERT-alerted
PE: Pulmonary Embolism
PP: Pre-PERT
PERT: Pulmonary Embolism Response Team
PESI: PE severity index
TTE: Transthoracic echocardiography
UVAMC: University of Virginia Medical Center
VTE: Venous Thromboembolism
