# Supplementary figures and images for: Adoption of a dedicated multidisciplinary team is associated with improved survival in acute pulmonary embolism
Source: Respir Res. 2020 Jun 22;21:159. doi: 10.1186/s12931-020-01422-z (PMC7310489; doi:10.1186/s12931-020-01422-z)

**Supplemental Figure 1**


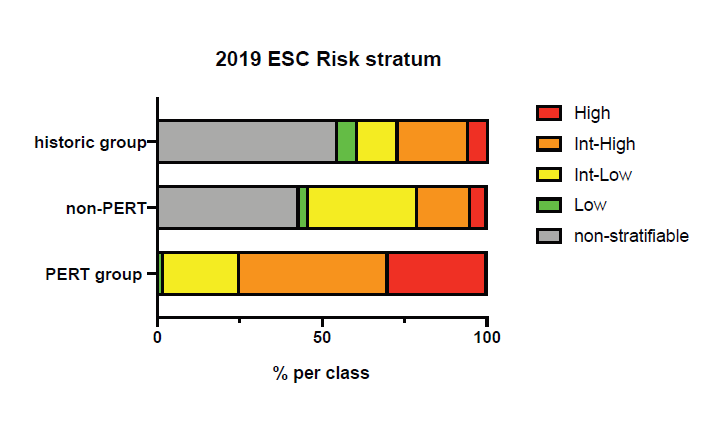

Supplement: Supplementary file 1 — Additional file 1: Supplemental Figure 1. Distribution of 2019 ESC risk categories among the 3 groups. ESC, European Society of Cardiology. [file 12931_2020_1422_MOESM1_ESM.docx]

**Supplemental Figure 2**


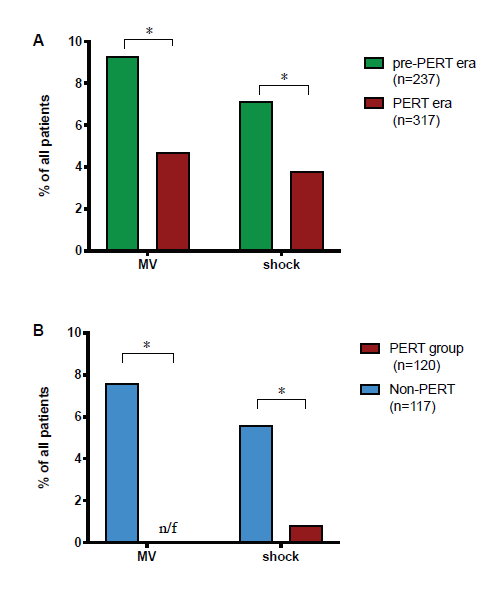

Supplement: Supplementary file 2 — Additional file 2: Supplemental Figure 2. Prevalence of non-PE related mechanical ventilation requirement and shock among patients among (A) pre-PERT and PERT-era patients and (B) Pert activated and non-PERT activated subjects in the PERT era; MV, mechanical ventilation, shock is defined as hypotension requiring vasopressor medication. * p < 0.001 by Chi square test. [file 12931_2020_1422_MOESM2_ESM.docx]
