# Supplementary material for: Adoption of a dedicated multidisciplinary team is associated with improved survival in acute pulmonary embolism
Source: Respir Res. 2020 Jun 22;21:159. doi: 10.1186/s12931-020-01422-z (PMC7310489; doi:10.1186/s12931-020-01422-z)
Supplement: Supplementary file 3 — Additional file 3: Supplementary Table 1 Performed risk assessment and severity of PE. Abbreviations: PP; pre-PERT; PA, PERT alerted; NPA, Non-PERT alerted; BNP, Brain natriuretic peptide; TTE, transthoracic echocardiogram; RV, right ventricle; CT, computer tomography. a) assessment within 12 h of pulmonary embolism diagnosis, b) positive RV strain by biomarkers was determined as troponin> 0.02 ng/mL, and/or BNP > 155 pg/mL; c) RV strain on CT imaging was determined as RV/LV ration (ratio of right ventricular to left ventricular diameter ration of > 0.9). P value calculated by Chi Square test. [file 12931_2020_1422_MOESM3_ESM.docx]

| **Supplementary table 1. Performed risk assessment and severity of PE** | | |  |  |
| --- | --- | --- | --- | --- |
|  | **pre-PERT (PP) (n=237)** | **PERT-alerted (PA) (n=120)** | **Non-PERT alerted (NPA) (n=197)** | ***P* value** |
| Assessment, No. (%) ^a)^ |  |  |  |  |
| Troponin? | 101 (43) | 119 (99) | 159 (81) | <0.001 |
| BNP? | 74 (31) | 119 (99) | 125 (64) | <0.001 |
| TTE? | 104 (44) | 98 (82) | 101 (51) | <0.001 |
| All 3? | 37 (16) | 97 (81) | 76 (39) | <0.001 |
| RV strain detected by, No. (%) |  |  |  |  |
| troponin ^b)^ | 94 (40) | 97 (81) | 110 (56) | <0.001 |
| TTE | 50 (21) | 80 (67) | 40 (20) | <0.001 |
| CT ^c)^ | 47 (20) | 65 (54) | 20 (10) | <0.001 |
| 2019 ESC risk category, No. (%) |  |  |  |  |
| Non-stratifiable | 128 (54) | 0 | 85 (43) | <0.001 |
| Low Risk | 14 (6) | 2 (2) | 5(3) | n.s. |
| Intermediate-Low Risk | 29 (12) | 28 (23) | 66 (34) | <0.01 |
| Intermediate -high Risk | 51 (22) | 54 (45) | 32 (16) | <0.01 |
| High Risk | 15 (6) | 36 (30) | 9 (5) | <0.001 |
